# Hepatocellular Carcinoma Surveillance Strategies: Major Guidelines and Screening Advances

**DOI:** 10.3390/cancers16233933

**Published:** 2024-11-24

**Authors:** Gavin Wu, Nojan Bajestani, Nooruddin Pracha, Cindy Chen, Mina S. Makary

**Affiliations:** Department of Radiology, The Ohio State University Wexner Medical Center, Columbus, OH 43210, USA; gavin.wu@osumc.edu (G.W.);

**Keywords:** hepatocellular carcinoma, HCC surveillance, liver cancer screening, imaging modalities, non-alcoholic fatty liver disease (NAFLD), biomarkers in HCC, early detection of HCC, APASL guidelines, AASLD guidelines, EASL–EORTC guidelines

## Abstract

Background/Objectives: Hepatocellular carcinoma (HCC) is a leading cause of cancer-related deaths globally, with prognosis and treatment outcomes that are significantly influenced by the stage at diagnosis. Early detection through regular surveillance is crucial for improving patient outcomes, especially in high-risk groups such as those with cirrhosis or chronic hepatitis B. Geographic variations in HCC risk factors, including viral hepatitis and non-alcoholic fatty liver disease (NAFLD), have led to the development of different international surveillance guidelines. This review aims to compare and evaluate the surveillance strategies proposed by the Asian Pacific Association for the Study of the Liver (APASL), the American Association for the Study of Liver Diseases (AASLD), and the European Association for the Study of the Liver and European Organization for Research and Treatment of Cancer (EASL–EORTC). Methods: The review examined and compared major international guidelines on HCC surveillance, focusing on patient selection, imaging modalities, and the integration of biomarkers. We also explored recent advancements in screening techniques, including artificial intelligence and emerging biomarkers, to identify future directions for improving surveillance strategies. Results: Our analysis identified key differences in the guidelines, particularly in imaging modality preferences and the use of biomarkers for early detection. While all guidelines place emphasis on high-risk populations, the inclusion criteria and surveillance intervals vary. Additionally, novel technologies such as artificial intelligence show potential to enhance the accuracy and efficiency of HCC detection. Conclusions: This review highlights the need to harmonize the international guidelines, particularly in regard to patients with non-cirrhotic NAFLD who remain under-represented in current surveillance protocols. Future research should focus on integrating emerging technologies and biomarkers to improve early detection and overall patient outcomes.

## Simple Summary

Hepatocellular carcinoma (HCC) is a leading cause of cancer-related deaths worldwide, and early detection plays a crucial role in improving patient survival. However, the current surveillance guidelines may not fully address the growing number of HCC cases in individuals with non-alcoholic fatty liver disease (NAFLD), who often develop cancer without liver cirrhosis. Our paper aims to review and compare major international guidelines on HCC surveillance, highlighting key gaps in their criteria such as the exclusion of non-cirrhotic NAFLD patients. By exploring advancements in screening technologies, including imaging and biomarkers, we aim to provide insights that could lead to better detection methods for high-risk populations. These findings have the potential to shape future research and updates to clinical guidelines, ensuring that surveillance strategies become more inclusive and effective.

## 1. Introduction

Hepatocellular carcinoma (HCC) is the most common type of primary liver cancer and the fifth most prevalent malignancy worldwide, with over 800,000 new cases diagnosed every year [1,2,3,4,5,6,7]. Chronic liver diseases, particularly cirrhosis and chronic hepatitis B virus (HBV), have historically been the primary risk factors for HCC, contributing to the high incidence of the disease in Sub-Saharan Africa and Asia [2,3,8,9]. However, there has been a significant rise in HCC cases linked to non-alcoholic fatty liver disease (NAFLD), especially in Western countries [3]. For instance, in the United States, where obesity and diabetes are prevalent, the incidence of HCC is projected to increase by 122% between 2016 and 2030 [3,8,10]. To address the rising global burden of HCC, it is crucial to examine current diagnostic strategies and enhance the surveillance of current and emerging risk factors.

HCC is the third leading cause of cancer-related mortality, with a five-year survival rate of 18% accounting for over 500,000 deaths annually [2,3,6,11]. Early detection of malignancy is crucial, as the prognosis is closely associated with the tumor stage [3,8]. For example, patients with early-stage HCC may benefit from curative treatments such as surgical resection and liver transplantation, with five-year survival rates exceeding 60% in some cases [3,8,12]. However, surgical resection is limited to patients classified as very early (Stage 0) or early (Stage A) according to the Barcelona Clinic Liver Cancer (BCLC) staging system, and patients seeking liver transplantation must meet the stringent Milan criteria of a single tumor ≤ 5 cm or up to three tumors with none > 3 cm, without macrovascular invasion or extrahepatic spread [3]. Unfortunately, less than 20% of patients qualify for these treatments due to late-stage diagnosis or advanced liver disease [12].

The often-late presentation of patients with HCC is generally attributed to the asymptomatic nature of the disease, with symptoms typically manifesting in the advanced stages [2,3]. These symptoms are more pronounced in patients with cirrhosis-related HCC, who often present with signs of decompensated liver failure such as worsening jaundice, ascites, and weight loss [3]. In contrast, patients with NAFLD-related HCC frequently lack cirrhosis, making detection more challenging [8,10].

The high morbidity and mortality rates of HCC, coupled with its asymptomatic progression until the advanced stages, underscores the need for effective surveillance to facilitate early detection at stages when curative treatments remain an option [1,3,8,12]. Given the geographical variations in HCC risk factors, such as the prevalence of viral hepatitis and NAFLD in certain regions, multiple international guidelines have been established to enhance surveillance strategies [3,8,10]. These guidelines, created by major organizations like the Asian Pacific Association for the Study of the Liver (APASL), the European Association for the Study of the Liver and European Organization for Research and Treatment of Cancer (EASL–EORTC), and the American Association for the Study of Liver Diseases (AASLD) offer evidence-based recommendations tailored to the specific needs of different populations [13,14,15]. As HCC continues to affect diverse populations worldwide, adapting to the shifting epidemiological landscape will be crucial for improving early detection and treatment outcomes [1,3,8,10].

This review will summarize and compare the HCC guidelines established by the APASL, EASL–EORTC, and the AASLD. Additionally, it will examine the imaging modalities and adjunctive techniques currently used in HCC surveillance, as well as recent advancements in screening strategies.

## 2. Methods

The data presented in this study are available in the Supplementary Materials file attached to this article. A comprehensive literature search was conducted using electronic databases, including PubMed, Embase, and Google Scholar, focusing on studies published from January 2008 to April 2024. Keywords included “hepatocellular carcinoma”, “HCC screening guidelines”, “biomarkers”, “ultrasound”, “APASL”, “AASLD”, “EASL”, and “HCC early detection”. Reference lists of relevant articles were manually reviewed to identify additional studies.

The inclusion criteria for this review were limited to studies and guidelines related to HCC surveillance, with a specific focus on imaging modalities such as ultrasound (US), contrast-enhanced ultrasound (CEUS), computed tomography (CT), and magnetic resonance imaging (MRI), as well as biomarkers like alpha-fetoprotein (AFP), des-gamma-carboxy prothrombin (DCP), and lectin-bound AFP (AFP-L3). Only peer-reviewed studies published in English were considered. Case reports, editorials, opinion pieces, and non-English publications were excluded. See Supplementary Figure S1 for a detailed PRISMA flowchart of study selection.

A total of 50 articles were identified by four independent reviewers. Data extraction involved summarizing key study characteristics, patient demographics, imaging and biomarker methods, and outcomes related to early HCC detection and surveillance strategies. A quality assessment of included studies is provided in Supplementary Table S1.

## 3. Overview of APASL, AASLD, and EASL–EORTC HCC Surveillance Guidelines

### 3.1. APASL Guidelines

#### 3.1.1. Surveillance Protocol

Initially introduced in 2010 and updated in 2016, the APASL guidelines for the surveillance, diagnosis, and management of HCC are specifically designed to address the unique epidemiological landscape of the Asia–Pacific region, where HBV infection remains a leading risk factor [5,14]. The recommended surveillance strategy involves a biannual combination of abdominal ultrasound (US) and serum AFP measurement [14]. This approach is intended to improve survival outcomes by identifying early-stage HCC, when potentially curative treatments such as resection or liver transplantation are most effective [14].

#### 3.1.2. Patient Selection Criteria

The APASL guidelines recommend HCC surveillance for high-risk individuals, including those with cirrhosis due to hepatitis B (HBV), hepatitis C (HCV), or non-alcoholic steatohepatitis (NASH) [14]. Surveillance is also advised for patients with genetic conditions such as hereditary hemochromatosis and Alpha-1 antitrypsin (A1AT) deficiency, as well as those with autoimmune liver disorders like primary biliary cirrhosis (PBC) and autoimmune hepatitis [14]. The guidelines also recommend HCC screening for patients on the liver transplant waitlist, regardless of liver function, as the identification of tumors that exceed the standard criteria is essential for determining transplant priorities [14]. A comprehensive list of high-risk groups as defined by APASL is provided in Table 1.

#### 3.1.3. Preferred Screening Tools

The APASL guidelines recommend a combination of abdominal US and serum AFP as the preferred screening tools for HCC surveillance, as they are cost-effective, non-invasive, and generally well tolerated by patients [14].

Dynamic computed tomography (CT) or magnetic resonance imaging (MRI) are not recommended as first-line screening tools due to a lack of clinical evidence in these modalities being used for HCC surveillance and their lack of cost-effectiveness [14]. However, the APASL guidelines advocate for the use of dynamic CT, MRI, contrast-enhanced ultrasound (CEUS), or gadolinium ethoxybenzyl diethylenetriamine pentaacetic acid (Gd-EOB-DTPA)-enhanced MRI as the primary diagnostic tools when an abnormal result is identified during HCC screening [14]. These imaging modalities provide a more comprehensive assessment of the liver and are better suited for confirming HCC diagnosis [14].

The APASL guidelines do not recommend the use of AFP or other serum biomarkers alone as confirmatory tests for HCC due to their limited sensitivity [14,16]. When used in combination with US for surveillance, an AFP cut-off value of 200 ng/mL is suggested [14,16]. However, in populations where hepatitis virus suppression or eradication has been achieved, a lower AFP cut-off value may be appropriate to enhance early detection [14,16].

#### 3.1.4. Criteria for Follow-Up and Referral

The APASL guidelines recommend that follow-up be based on certain imaging characteristics [14]. If nodular lesions show atypical imaging features, such as iso- or hypovascularity in the arterial phase or hypervascularity without portal venous washout, further evaluation using CT, MRI, or CEUS is necessary to confirm the diagnosis and rule out other conditions [14].

### 3.2. AASLD Guidelines

#### 3.2.1. Surveillance Protocol

First established in 2005, with subsequent revisions made in 2010 and 2018, the AASLD guidelines emphasize the importance of surveillance in patients with cirrhosis, regardless of the underlying cause [5,15,17]. The recommended surveillance protocol involves a semi-annual liver US combined with AFP testing, conducted at approximately six-month intervals [15]. To enhance surveillance, utilization, and patient adherence, the guidelines also advocate for outreach interventions, including provider education, mailed surveillance invitations, and electronic medical record reminders [15].

#### 3.2.2. Patient Selection Criteria

The AASLD guidelines recommend HCC surveillance for patients at high risk of developing malignancy and those that are eligible for treatment [15]. This group includes individuals with Child-Pugh stage A or B cirrhosis, as well as those with Child-Pugh stage C cirrhosis who are candidates for liver transplantation [15]. All patients listed for liver transplantation are also advised to undergo semi-annual surveillance, as early HCC detection can impact their transplantation priority [15].

The AASLD guidelines also outline specific patient groups for whom routine surveillance is not recommended [15]. This includes patients with Child-Pugh class C cirrhosis who are not eligible for liver transplantation, those with life-limiting comorbidities that cannot be addressed by liver transplantation or other treatments, and patients with advanced fibrosis due to HCV or NAFLD who do not have cirrhosis [15].

#### 3.2.3. Preferred Screening Tools

The AASLD recommends abdominal US combined with AFP as the preferred screening tools for HCC surveillance, citing both cost-effectiveness and improved diagnostic accuracy when both tools are employed together [15].

The AASLD currently advises against using CT or MRI for routine HCC surveillance in at-risk patients [15]. This recommendation stems from concerns over repeated radiation and contrast exposure with CT, as well as the high costs and lack of patient acceptance with MRI [15].

In addition, other serum biomarkers are currently not recommended for routine surveillance due to insufficient data supporting their clinical efficacy [15].

#### 3.2.4. Criteria for Follow-Up and Referral

Follow-up and referral procedures are based on US findings, the presence of liver lesions, and AFP levels. If the US findings demonstrate adequate visualization (LI-RADS score A) with no liver lesions and normal AFP levels, patients should continue semi-annual surveillance with US and AFP. For patients with limited US visibility, the AASLD guidelines recommend using contrast-enhanced MRI or multiphase CT for surveillance.

In cases where a subcentimeter liver lesion is detected, a repeat US and AFP test in 3–6 months is recommended due to the low risk of HCC and the limited diagnostic accuracy of CT or MRI for lesions < 1 cm. If the lesion remains stable after two or more follow-ups, the patient can return to regular surveillance.

For new or enlarging liver lesions ≥ 1 cm, the AASLD guidelines recommend diagnostic imaging with multiphase contrast-enhanced CT or MRI. Further imaging may also be initiated in patients with serum AFP levels ≥ 20 ng/m, rising AFP levels across consecutive tests, or doubling AFP levels. For patients with markedly elevated AFP levels (≥200 ng/mL) but no detectable liver mass, additional imaging including PET-CT may be necessary to identify the underlying cause.

### 3.3. EASL–EORTC Guidelines

#### 3.3.1. Surveillance Protocol

The EASL–EORTC first introduced guidelines for the surveillance and management of HCC in 2005, with significant updates in 2008 and 2012 to reflect advancements in clinical practice [5,13]. These guidelines emphasize the importance of HCC surveillance not only for patients with cirrhosis, but also for high-risk individuals without cirrhosis such as those with non-cirrhotic HBV infections [13]. For these high-risk populations, the EASL–EORTC guidelines recommend that surveillance be conducted every six months using abdominal US [13].

#### 3.3.2. Patient Selection Criteria

The EASL–EORTC guidelines for HCC surveillance prioritize a cost-effective approach, recommending specific patient groups for regular monitoring based on their risk profiles [13]. Surveillance is recommended for cirrhotic patients with Child-Pugh stage A and B, given their elevated risk of developing HCC [13]. Additionally, cirrhotic patients with Child-Pugh stage C who are awaiting liver transplantation should also undergo surveillance, as early detection of HCC is crucial for managing their transplant eligibility [13].

The EASL–EORTC guidelines extend surveillance recommendations to patients without cirrhosis, including those with chronic HBV infection who are classified as being at intermediate or high risk for HCC [13]. Furthermore, non-cirrhotic patients with advanced fibrosis, regardless of the underlying etiology, may also be considered for surveillance based on an individual risk assessment [13].

#### 3.3.3. Preferred Screening Tools

The EASL–EORTC guidelines recommend abdominal US as the preferred screening tool for HCC surveillance due to its non-invasiveness, absence of risks, patient acceptance, moderate cost, and ability to detect other cirrhosis-related complications such as subclinical ascites or portal vein thrombosis [13]. The guidelines also emphasize the importance of having experienced personnel conduct the surveillance, as the effectiveness of US in early HCC detection is highly dependent on the operator’s technique [13].

The EASL–EORTC guidelines advise against using CT and MRI as screening tools for HCC surveillance due to their general lack of cost-effectiveness and high rates of false positives [13]. In addition, these imaging modalities pose potential risks to patients, such as repeated radiation exposure from CT scans, high costs associated with MRIs, and the risk of allergic reactions to contrast agents [13]. Despite these limitations, CT and MRI may be considered as alternatives to US for patients on liver transplant waiting lists or when US is hindered anatomically, such as in obese patients or those with chest wall deformities [13].

The EASL–EORTC guidelines do not currently recommend tumor biomarkers such as AFP, AFP-L3, and DCP for routine early HCC detection, as they lack cost-effectiveness and sufficient clinical efficacy [13].

#### 3.3.4. Criteria for Follow-Up and Referral

For follow-up and referral criteria, the EASL–EORTC guidelines emphasize a structured approach based on nodule size and patient risk [13]. Abnormal US results such as newly detected focal lesions or known lesions with echo pattern changes or enlargement should prompt further investigation [13]. For high-risk patients, nodules smaller than 1 cm detected by US should be monitored at intervals of ≤4 months during the first year [13]. If no increase in the size or number of nodules is observed, the surveillance interval can be extended to the standard six months [13].

Any nodule larger than 1 cm should be considered abnormal and trigger further diagnostic efforts, utilizing non-invasive imaging criteria such as CT or MRI [13]. If non-invasive criteria are inconclusive, biopsy is warranted [13]. If the biopsy results are non-diagnostic or discordant with imaging, strict follow-up every 3–4 months is recommended [13]. Upon the detection of suspicious nodule, the EASL–EORTC guidelines also recommend evaluation at a specialized referral center with the appropriate expertise and resources [13].

### 3.4. Comparative Analysis of HCC Screening Guidelines

As discussed throughout our review, surveillance plays a pivotal role in HCC management, as early detection allows for the identification of small, asymptomatic tumors that may be treated with curative options such as resection or liver transplantation [3]. Current guidelines from the APASL, EASL–EORTC, and AASLD recommend regular surveillance in at-risk populations, typically every six months, using US with or without alpha-fetoprotein (AFP) testing [1,5,8,13,14,15]. While these guidelines share many common strategies, they also differ in their approaches to patient selection, screening modalities, and criteria for follow-up and referral. The key similarities and differences between these guidelines, summarized in Table 2, will be discussed below.

#### 3.4.1. Surveillance Protocol

The APASL, AASLD, and EASL–EORTC guidelines all recommend HCC surveillance using abdominal US at six-month intervals. This timing is supported by clinical evidence which shows that semi-annual screening effectively balances the advantages of early detection with the risks and costs associated with more frequent testing. All three guidelines emphasize a six-month interval as the optimum for maximizing early HCC detection while reducing unnecessary interventions [13,14,15,16].

#### 3.4.2. Patient Selection Criteria

Regarding patient selection criteria, the APASL, AASLD, and EASL–EORTC guidelines share a common focus on targeting high-risk populations for screening, but they differ in how broadly they define these high-risk groups. All three guidelines emphasize the importance of regular surveillance for patients with cirrhosis, especially those in Child-Pugh stages A and B, due to their increased risk of developing HCC. Additionally, they recommend HCC surveillance for patients awaiting liver transplantation, as early detection can significantly impact transplant eligibility and prioritization [13,14,15,16].

However, the guidelines diverge in their inclusion of other high-risk groups, reflecting their varying approaches to balancing the benefits of surveillance with cost-effectiveness [13,14,15,16]. For instance, the AASLD guidelines take a more conservative stance, recommending surveillance only for patients who have cirrhosis and are eligible for treatment. This includes patients with Child-Pugh stage A or B cirrhosis, and transplant candidates with Child-Pugh stage C cirrhosis [15].

They advise against routine surveillance for patients with Child-Pugh stage C cirrhosis who are not transplant candidates and those with significant comorbidities [15]. In contrast, the APASL guidelines adopt a broader approach, recommending surveillance not only for cirrhotic patients but also for those with certain genetic conditions, autoimmune liver disorders, and NASH [14]. The EASL–EORTC guidelines also extend surveillance to non-cirrhotic patients with chronic HBV infection or advanced fibrosis, although they suggest individual risk assessments for such patients.

#### 3.4.3. Preferred Screening Tools

The APASL, AASLD, and EASL–EORTC guidelines recommend abdominal US as the primary screening tool for HCC surveillance, highlighting its non-invasive nature, widespread availability, cost-effectiveness, and overall patient tolerability [13,14,15,16]. However, the guidelines differ in their recommendations regarding the use of AFP as a supplementary biomarker. The APASL guidelines strongly advocate for combining AFP with US in surveillance protocols, citing improved diagnostic sensitivity [14]. Similarly, the AASLD supports the use of AFP, acknowledging its potential to enhance early detection rates when paired with US [15]. In contrast, the EASL–EORTC guidelines advise against routine AFP testing, citing concerns over cost-effectiveness and insufficient clinical evidence supporting its use in routine surveillance [13].

#### 3.4.4. Criteria for Follow-Up and Referral

##### Preferred Imaging Modalities

The APASL, AASLD, and EASL–EORTC guidelines all emphasize the importance of thorough follow-up and referral procedures when abnormal findings are detected during HCC surveillance. If nodules display unusual characteristics or exceed specific size thresholds, all three guidelines recommend further evaluation with CT or MRI for a comprehensive liver assessment [13,14,15,16]. However, their recommendations regarding CEUS differ [17].

The APASL guidelines consider CEUS to be as sensitive as CT or MRI, while the EASL–EORTC guidelines also support the use of CEUS for diagnosing nodules ≥ 1 cm in cirrhotic patients [13,14,17]. In contrast, the AASLD does not endorse the routine use of CEUS due to concerns regarding operator dependency, variability in visualization based on patient and tumor factors, and its limited ability to provide staging information which is critical for treatment planning [15,17]. Nevertheless, the AASLD recognizes CEUS as a secondary option when MRI or CT are inconclusive, unavailable, or contraindicated, especially in cases where a tumor biopsy cannot be performed [15].

##### Management of Suspicious Nodules

The guidelines also differ in their approach to managing suspicious nodules and the subsequent diagnostic steps [13,14,15,17]. The APASL guidelines prioritize imaging characteristics for HCC diagnosis, recommending further evaluation for nodules with atypical features such as iso- or hypovascularity, or hypervascularity without portal venous washout [14,18]. In contrast, the AASLD guidelines incorporate both US findings and serum AFP levels, advising repeat imaging in 3–6 months for subcentimeter lesions and recommending multiphase CT or MRI for patients with lesions ≥ 1 cm or elevated AFP levels [15]. They also propose PET-CT for patients with very high AFP levels but no detectable mass [15].

The EASL–EORTC guidelines recommend more frequent monitoring for nodules smaller than 1 cm in high-risk patients, with follow-up imaging every 3–4 months [13]. For nodules larger than 1 cm, further evaluation with CT or MRI is required [13]. If these imaging results remain unclear, a liver biopsy is necessary, with continued intensive follow-up every 3 to 4 months if the biopsy results are inconclusive or contradictory to the imaging findings [13]. Compared to the broader follow-up strategies of the APASL and AASLD, the EASL–EORTC guidelines emphasize the need for specialized referral centers to effectively manage suspicious nodules [13,14,15].

## 4. Imaging Modalities

Imaging modalities play a critical role in the screening and early detection of HCC, particularly in high-risk populations where early intervention can greatly improve the outcomes. The most commonly used imaging techniques in clinical practice include US, CEUS, MRI, and CT, each offering unique advantages and limitations. These modalities not only aid in early diagnosis but also serve as key components in surveillance algorithms, guiding subsequent diagnostic and therapeutic decisions. As technology evolves, understanding the strengths and limitations of each modality is crucial for optimizing HCC surveillance strategies.

### 4.1. Ultrasound and Contrast-Enhanced Ultrasound

US is the most commonly used modality for HCC screening due to its non-invasiveness, cost-effectiveness, and widespread availability [13,14,15,18]. It is typically performed every six months in high-risk patients, such as those with cirrhosis or chronic hepatitis B, enabling the detection of small, asymptomatic tumors [19]. Despite its frequent use in surveillance, US has limitations including sensitivity ranging from 40–81% and specificity from 80–100% [3]. Its effectiveness is highly dependent on operators’ skills and can be compromised in obese patients or those with advanced cirrhosis, where altered liver echotexture may obscure small lesions [13]. In such cases, more advanced imaging techniques are often required for follow-up when the US results are inconclusive [13].

To address some of the limitations of conventional US, CEUS has emerged as a valuable complementary tool. CEUS improves the capability of conventional US by using microbubble contrast agents to enhance the visualization of blood flow and tumor vascularity [14]. CEUS is particularly effective for characterizing liver nodules detected on US and is comparable in sensitivity to dynamic CT or MRI for diagnosing HCC [14]. It can detect critical hemodynamic changes during hepatocarcinogenesis, such as increased arterial flow and decreased portal flow which are essential for diagnosing HCC [14]. CEUS also offers several advantages over other modalities, including being relatively inexpensive, non-nephrotoxic, and free from ionizing radiation [14].

Despite these benefits, the AASLD removed CEUS from its guidelines due to concerns about false-positive diagnoses of HCC in patients with intrahepatic cholangiocarcinoma [15]. However, emerging evidence has shown that with specific timing criteria for contrast washout, CEUS can achieve high diagnostic accuracy [14,20]. In addition, once a liver lesion is detected on US, CEUS is considered a cost-effective second-line imaging modality [14]. Despite its utility, CT or MRI remain the gold standard for characterizing small nodules at that are high risk for HCC, particularly in Western guidelines [3,18].

### 4.2. CT and MRI

CT and MRI play a crucial role in the diagnostic workup of HCC, particularly when abnormalities are detected during an initial screening. These advanced imaging modalities not only aid in diagnosing HCC, but also inform critical clinical decisions such as treatment plans or eligibility for surgery [13,14,15]. Dynamic CT and MRI, especially with gadolinium-based contrast agents such as Gd-EOB-DTPA, are recommended as first-line diagnostic tools when an initial screening test yields abnormal results [3]. These modalities provide detailed imaging that captures the hallmark features of HCC, such as arterial phase enhancement followed by washout in the portal venous and/or delayed phases, offering high sensitivity and specificity for diagnosing HCC in high-risk populations [14,20].

Dynamic CT and MRI play a pivotal role in accurate staging and treatment planning due to their ability to precisely visualize the vascularity of liver lesions. The sensitivity of MRI for detecting nodular HCC ranges from 77–100%, while CT shows a slightly lower sensitivity between 68–91% [3,21]. MRI, particularly when using Gd-EOB-DTPA, has demonstrated superior sensitivity in the detection of small tumors compared to CT and is increasingly favored for its ability to identify early HCC through specific imaging biomarkers such as hypointensity on hepatobiliary phase images [19].

Combining dynamic and hepatobiliary phase imaging with diffusion-weighted imaging (DWI) can further enhance the diagnostic accuracy of MRI in detecting HCC. However, certain challenges remain such as the potential for ‘pseudo-washout’ during the transitional phase of Gd-EOB-DTPA-enhanced MRI, which may reduce the specificity of HCC diagnosis [3]. Additionally, while MRI demonstrates excellent sensitivity for identifying small HCCs, it can have difficulty distinguishing between early HCC and high-grade dysplastic nodules, highlighting the need for further studies to refine non-invasive diagnostic criteria [3]. The availability and cost of MRI may also limit its routine use in surveillance, particularly in resource-limited settings [13,14,15].

### 4.3. Guideline Recommendations for Imaging Modalities

As previously discussed, the APASL, AASLD, and EASL–EORTC guidelines exhibit notable similarities in their approaches to HCC screening despite the differences in regional epidemiology and resource availability. The AASLD guidelines recommend US as the primary screening modality, with CEUS, MRI, or CT reserved for cases where US is insufficient or detects suspicious lesions [21]. Similarly, the EASL–EORTC guidelines prioritize US but advise MRI or CT for further evaluation when US findings are inconclusive [13]. The APASL guidelines also emphasize US for routine surveillance, supporting the use of CT or MRI in high-risk patients or when US results are indeterminate [14,21]. This consensus across the guidelines reinforces the importance of early detection via US, while advanced imaging techniques are reserved for cases requiring detailed lesion characterization or when initial screening results are unclear.

In summary, while US remains the cornerstone of HCC screening across all major guidelines due to its accessibility and cost-effectiveness, CEUS, CT, and MRI play essential roles in further characterizing and confirming HCC. As recommended by the guidelines, imaging modality selection should be tailored to patient-specific needs, available resources, and clinical contexts, ensuring that HCC surveillance and diagnosis remain both effective and adaptable across diverse healthcare settings. A detailed summary of the primary uses, guideline recommendations, and key aspects of each imaging modality is provided in Table 3.

## 5. Adjunctive Techniques

Biomarkers have become an important component of HCC screening strategies, particularly when used alongside imaging modalities such as US. Their primary role is to enhance detection and risk stratification in patients at risk of developing HCC, facilitating earlier diagnosis and more effective management. While imaging remains the cornerstone of HCC detection, biomarkers like AFP add diagnostic value, especially in cases where imaging results are inconclusive or in high-risk patients such as those with chronic hepatitis B or cirrhosis. Integrating biomarkers into HCC screening protocols improves the sensitivity and specificity of detection, potentially leading to better outcomes through earlier intervention [3].

### 5.1. Common Biomarkers

Among the various biomarkers used in HCC screening, AFP is the most widely recognized and utilized. AFP is a glycoprotein produced by the fetal liver, with levels typically declining after birth. In adults, elevated AFP levels may indicate HCC, especially when exceeding 200 ng/mL [3,14]. However, the sensitivity and specificity of AFP alone are insufficient for it to serve as a standalone diagnostic tool, as elevated AFP can also be observed in patients with other liver conditions such as chronic hepatitis and cirrhosis. Despite these limitations, AFP remains a valuable adjunctive tool in HCC surveillance, particularly when combined with imaging modalities like US. This combination aids in the identification of patients who require closer monitoring or further diagnostic evaluation [3].

### 5.2. Emerging Biomarkers

In addition to AFP, emerging biomarkers such as des-gamma-carboxy prothrombin (DCP) and lectin-bound AFP (AFP-L3) are being investigated for their potential to improve diagnostic accuracy in HCC screening [3,22]. DCP, also known as PIVKA-II, is an abnormal form of prothrombin that is elevated in HCC patients, particularly those with normal AFP levels [22]. It is more commonly utilized in Asia and is included in the APASL guidelines for HCC screening [14,23]. AFP-L3, a glycoform of AFP, offers greater specificity for HCC and is particularly useful in distinguishing HCC from benign liver conditions in patients with elevated total AFP levels [14,23,24].

Several additional biomarkers have demonstrated potential in HCC screening and are currently being studied for their diagnostic value. Golgi Protein 73 (GP73), a membrane protein that shows elevated levels in patients with HCC, has emerged as a notable marker [22]. In some studies, GP73 has been found to exhibit higher sensitivity than AFP, suggesting that it could be a valuable tool for early detection, particularly in patients where AFP may fall short [22,25]. Osteopontin (OPN), another glycoprotein that is elevated in various cancers, including HCC, has gained attention for its ability to distinguish between HCC and benign liver diseases, which may help reduce false-positive diagnoses [26]. Additionally, Glypican-3 (GPC3), a heparan sulfate proteoglycan that is overexpressed in HCC, plays a key role in tissue-based diagnosis [22,25]. Its utility is particularly evident when used in conjunction with immunohistochemistry, helping to enhance diagnostic accuracy, especially in histological evaluations of liver lesions [22]. A summary of these biomarkers is provided in Table 4.

### 5.3. Guideline Recommendations for Biomarkers

Recommendations for biomarker use in HCC screening vary across clinical guidelines, reflecting differences in regional practices and epidemiology. For instance, while the AASLD guidelines recommend the use of AFP alongside US, particularly in high-risk patients or in settings where imaging quality is compromised, they advise against AFP as a standalone screening tool due to its limited sensitivity and specificity [15]. Similarly, the EASL–EORTC guidelines acknowledge the role of combining AFP with imaging in HCC surveillance, rather than relying on it as a primary screening method [13]. This combined approach highlights the importance of integrating biomarkers with imaging to improve early detection rates and ensure that high-risk patients receive appropriate monitoring [13]. In contrast, the APASL guidelines place greater emphasis on the use of biomarkers in HCC screening. They support the integration of biomarkers into routine screening protocols to enhance the sensitivity of surveillance, particularly in high-risk populations such as those with chronic hepatitis B or cirrhosis [14]. These differing approaches reflect the evolving role of biomarkers in HCC surveillance, with each association tailoring its recommendations to the unique needs of its population.

## 6. Discussion

### 6.1. NAFLD and HCC: Current Challenges and Preventive Strategies

#### 6.1.1. Gaps and Limitations in Existing Guidelines

NAFLD encompasses a broad spectrum of liver conditions characterized by the accumulation of fat in hepatocytes in individuals with no significant alcohol consumption [3]. NAFLD ranges from a non-alcoholic fatty liver (NAFL), which involves simple hepatic steatosis without inflammation, to non-alcoholic steatohepatitis (NASH), a more severe form marked by steatosis, inflammation, and hepatocellular injury [31]. NAFLD is strongly associated with metabolic syndrome, which includes conditions such as hypertension, dyslipidemia, insulin resistance, and obesity [32]. As these risk factors continue to rise globally, NAFLD has become one of the most common liver diseases, affecting an estimated 25% of the global population. Projections suggest that the incidence of NAFLD could increase by as much as 56% by 2030, further highlighting its growing public health burden [31,32].

NAFLD has become a leading cause of HCC, particularly in Western countries where obesity, diabetes, and other metabolic conditions are endemic [32]. Compared to HCC caused by other factors such as viral hepatitis, NAFLD-associated HCC is often diagnosed at a more advanced stage and is associated with poorer survival outcomes [32]. This may be attributed to the fact that NAFLD-related HCC often develops in the absence of cirrhosis, with up to one-third of cases occurring without fibrosis [14,15,32]. Unfortunately, current surveillance guidelines from major organizations such as the APASL, AASLD, and EASL–EORTC do not include specific recommendations for patients with non-cirrhotic NAFLD [13,14,15]. This gap in surveillance may contribute to the under-recognition of the disease, delayed diagnoses, and poorer outcomes [32]. As the global prevalence of obesity, diabetes, and other NAFLD-associated risk factors continues to rise, the burden of NAFLD-related HCC is expected to increase, highlighting the urgent need for updated surveillance protocols that address this high-risk population [14].

#### 6.1.2. Current Guideline Recommendations

As previously discussed, the current guidelines provide recommendations for HCC surveillance in patients with cirrhosis, including those with cirrhosis caused by NAFLD, but they do not offer specific surveillance recommendations for patients with non-cirrhotic NAFLD. Despite this, all three guidelines—from the APASL, AASLD, and EASL–EORTC—acknowledge the rising prevalence of metabolic syndrome and obesity, and their strong association with the increasing incidence of NAFLD [13,14,15]. For instance, the EASL–EORTC guidelines highlight the growing burden of NAFLD-induced HCC in developed countries such as the United States, where over 500,000 HCC cases are expected, largely driven by the high prevalence of metabolic syndrome [13]. The guidelines further recognize that NAFLD can progress to HCC even in the absence of cirrhosis. Most importantly, the APASL guidelines emphasize the critical gap in current surveillance protocols, which fail to address the increasing number of non-cirrhotic HCC cases [14].

Despite their acknowledgement of the increasing prevalence of non-cirrhotic NAFLD and its potential to progress to HCC, none of the three major guidelines currently endorse HCC surveillance for this patient population [13,14,15]. For instance, the APASL guidelines do not offer any recommendations for HCC surveillance in non-cirrhotic NAFLD patients, citing the need for more evidence before a formal strategy can be implemented [14]. Similarly, the AASLD guidelines advise against surveillance in patients with advanced fibrosis without cirrhosis, including those with non-cirrhotic NAFLD, due to concerns over cost-effectiveness [15]. The EASL–EORTC guidelines also advise against surveillance in this population, reasoning that the rate of progression to HCC in non-cirrhotic NAFLD patients is not high enough to justify the implementation of surveillance programs [13]. Furthermore, they note that obesity may limit the effectiveness of routine US screening in many of these patients, necessitating more expensive imaging modalities such as CT or MRI [13]. Additionally, significant comorbidities such as cardiovascular disease may render these patients ineligible for surgery, thus diminishing the potential benefits of surveillance altogether [13].

#### 6.1.3. Future Directions

Given the increasing global burden of NAFLD, there is an urgent need to re-evaluate and expand the current surveillance guidelines to include non-cirrhotic patients who are at high risk of developing HCC. By addressing these gaps, early detection efforts could be significantly improved, leading to better clinical outcomes for this expanding high-risk population.

However, current evidence supporting surveillance in non-cirrhotic NAFLD patients remains conflicting. For example, while the APASL acknowledges emerging evidence that suggests an increased incidence of HCC in this group, the AASLD guidelines argue that existing clinical data show a very low annual incidence of HCC in these patients [14,15]. Given these uncertainties, all three guidelines emphasize the need for further research, including obtaining more data from cohorts of patients with NASH or NAFLD to better identify high-risk patients who would benefit from surveillance [13]. Additionally, more studies are required to accurately estimate the risk of HCC development in non-cirrhotic NAFLD patients [13].

According to the guidelines, this focus on identifying high-risk patients serves as a potential strategy for determining who should be screened for HCC [13]. For instance, while the AASLD guidelines acknowledge the need for more data, they also suggest that risk stratification tools could eventually aid in selecting those at the highest risk for targeted surveillance [15]. In the interim, surveillance may be considered on a case-by-case basis for select patients with advanced fibrosis [15].

Emerging biomarkers and laboratory markers may also help identify high-risk patients within the NAFLD population. Several clinical studies have shown that the PNPLA3 148M variant at rs738409, commonly found in obese patients with NAFLD, may be linked to an increased risk of HCC development [10,13]. However, further research is required to assess whether these findings can significantly contribute to stratifying NAFLD patients at risk for HCC and guide surveillance strategies [13].

Despite the need for further research, all three guidelines acknowledge the increased risk of HCC in patients with risk factors like metabolic syndrome and obesity. Consequently, the guidelines strongly recommend preventive measures targeting these comorbidities, emphasizing lifestyle interventions such as dietary modifications and regular exercise [13,14].

### 6.2. The Role of Biomarkers in Risk Stratification and Surveillance

#### 6.2.1. Gaps and Limitations in Existing Guidelines

As previously mentioned, biomarkers present a potentially cost-effective and non-invasive option for HCC surveillance. The most widely used biomarker, AFP, is endorsed by two international organizations—the AASLD and EASL–EORTC—for use alongside US to improve detection. However, its current role in screening protocols is limited by its low sensitivity and restricted availability. For instance, AFP’s sensitivity to early-stage HCC remains relatively low, at around 40–60%, leading to a substantial number of missed cases, particularly in the early stage of disease [33]. Moreover, AFP levels can be falsely elevated in patients with chronic liver disease or viral hepatitis, resulting in a notable rate of false positives [34].

Another significant challenge is the biological heterogeneity of HCC, which affects AFP’s reliability as a biomarker. Not all HCC tumors secrete AFP, and its levels can fluctuate depending on the tumor size, grade, and the presence of underlying liver disease [35]. This variability limits AFP’s effectiveness in detecting early-stage or well-differentiated tumors, where levels may remain low. Additionally, the heterogeneity of HCC suggests that other tumor markers might be more valuable for specific subtypes. For instance, AFP is often less reliable in small or slow-growing tumors, potentially delaying diagnosis until the disease reaches more advanced stages [36].

The cost-effectiveness of incorporating biomarkers into routine surveillance is also a topic of debate, considering their limited performance and the added expense of frequent testing [13]. As a result, current guidelines remain cautious, favoring imaging-based methods as the primary surveillance tool [13,14,15]. However, adopting a multi-biomarker approach in future strategies could mitigate these limitations by enhancing sensitivity and offering more personalized screening options that account for the heterogeneity of HCC tumors.

#### 6.2.2. Future Directions

Nonetheless, emerging research into biomarkers has shown their promising potential for enhancing HCC detection and risk stratification. Advancements in laboratory techniques and the broader availability of biomarker assays hold great promise for making these tools more cost-effective and valuable as part of a multi-modal HCC screening strategy. For instance, when used alongside US, biomarkers have the potential to improve the sensitivity and specificity of early HCC detection. The APASL guidelines have already recognized the importance of adjusting AFP cut-off values based on population-specific factors, especially in regions where hepatitis virus suppression or eradication has been achieved [14]. In these populations, lowering the AFP cut-off value could enhance the early detection of HCC, enabling more timely interventions [14]. This adaptive approach to AFP thresholds, tailored to specific epidemiological and virological profiles, may significantly improve the utility of AFP when combined with US [33]. Future guidelines could build on this by refining AFP and other biomarker cut-off values to account for factors like the cirrhosis stage, viral load, and patient demographics. This personalized approach would overcome the limitations of a ‘one-size-fits-all’ model and support more targeted screening.

As discussed previously, alternative biomarkers such as des-gamma-carboxy prothrombin (DCP) and AFP-L3 have demonstrated potential for improving HCC detection in specific cases. DCP is particularly promising in more advanced cases or in patients with normal AFP levels. Although its sensitivity to early-stage HCC remains suboptimal, its greater specificity compared to AFP could help reduce missed cases [37]. AFP-L3, a glycoform of AFP, is associated with more aggressive tumor behavior and may help stratify high-risk patients. The integration of AFP-L3 into surveillance algorithms could enable more personalized management based on tumor biology and aggressiveness [37]. However, despite their potential, these biomarkers have yet to be widely adopted into current surveillance guidelines due to their limited validation and inconsistent performance in detecting early-stage HCC.

In addition to AFP, DCP, and AFP-L3, emerging biomarkers such as microRNAs (miRNAs) and circulating tumor DNA (ctDNA) are gaining attention in HCC research. miRNAs play critical roles in tumorigenesis and have been identified as potential diagnostic and prognostic markers for HCC. Specific miRNAs such as miR-21 and miR-122 have shown good diagnostic accuracy in early-stage HCC, potentially surpassing AFP in sensitivity [38]. Similarly, ctDNA offers a non-invasive method for detecting tumor-specific genetic alterations, providing real-time insights into tumor evolution and recurrence risk [39]. Incorporating these advanced biomarkers into future guidelines could significantly enhance the early detection and monitoring of HCC [39].

Recent advancements in HCC biology have also paved the way for novel screening techniques such as liquid biopsies, which allow for the detection of circulating tumor DNA (ctDNA), circulating tumor cells (CTCs), and other tumor-derived components in blood samples. These non-invasive methods offer a powerful tool for early cancer detection and monitoring, potentially identifying tumors that are undetectable on imaging or traditional serum markers. While they are still under investigation, these approaches could revolutionize HCC screening by providing more personalized and accurate diagnostic information, particularly in high-risk populations [40,41].

Additionally, integrating artificial intelligence (AI) and machine learning into HCC screening holds significant potential. AI-driven algorithms can analyze large datasets of imaging and biomarker data, identifying patterns and risk factors that may be overlooked by human observers. These technologies could improve the sensitivity and specificity of HCC screening, particularly in diverse populations with varying risk profiles and healthcare access. As these innovations evolve, they are likely to be incorporated into future guidelines, offering more refined and individualized screening strategies with the potential to improve patient outcomes [40,42].

Although further research is needed, future updates to surveillance guidelines could benefit from incorporating a multi-biomarker approach, integrating established markers like AFP with newer candidates such as DCP, AFP-L3, miRNAs, and ctDNA. This approach could enhance diagnostic accuracy, facilitate the earlier detection of HCC in high-risk populations, and support more personalized screening protocols.

### 6.3. Considerations for Low-Risk Populations

#### 6.3.1. Gaps and Limitations in Existing Guidelines

Current HCC surveillance guidelines primarily focus on high-risk populations such as individuals with cirrhosis or advanced fibrosis, but often lack specific recommendations for the management of low-risk populations. The existing approaches frequently do not consider individual risk factors beyond cirrhosis and fibrosis, such as genetic predispositions, lifestyle factors, and specific biomarkers. This lack of stratification creates a substantial gap in the guidance, potentially leading to overdiagnosis and overtreatment, which can result in unnecessary interventions and significant financial burdens for individuals with minimal HCC risk. For example, the broad application of surveillance to non-cirrhotic NAFLD patients, who are at a relatively low risk of developing HCC, may increase healthcare costs due to incidental findings or false positives, with minimal clinical benefit. As such, balancing cost-effectiveness with clinical benefit is a critical challenge in these groups, as extensive surveillance may yield a high number of screenings relative to the small number of cases detected [10,43,44,45].

#### 6.3.2. Future Directions

Refining HCC surveillance guidelines to address low-risk populations requires a personalized, risk-based approach. Future directions in HCC surveillance should focus on developing more nuanced models that integrate clinical, genetic, and molecular factors to better stratify risk and tailor surveillance strategies [43,45,46,47,48]. For example, incorporating genetic markers and lifestyle factors into risk models can enhance the precision of HCC surveillance, making it both more cost-effective and clinically relevant. Such tools could help identify subsets of low-risk individuals who may be safely excluded from routine surveillance, thereby reducing the risks of overdiagnosis and overtreatment while optimizing resource allocation and improving clinical outcomes [46,49,50].

In addition, cost-effectiveness analyses are playing an increasingly pivotal role in shaping future HCC surveillance guidelines. By evaluating the balance between surveillance costs and HCC detection rates in low-risk populations, health policy-makers and clinicians can make more informed decisions about resource allocation. Establishing risk thresholds that justify surveillance would improve guideline precision and focus efforts on the individuals most likely to benefit. Ultimately, integrating evidence-based criteria into HCC surveillance protocols may facilitate more efficient, personalized care that aligns with global trends in precision medicine and oncology [10].

## 7. Conclusions

Our review of HCC surveillance guidelines from the APASL, AASLD, and EASL–EORTC highlights key elements regarding screening protocols, imaging modalities, and the role of adjunctive techniques in surveillance. All three guidelines recommend biannual US surveillance, with some advocating for the addition of AFP testing to enhance early detection in high-risk populations such as those with cirrhosis. Notably, the APASL and AASLD guidelines recommend the use of AFP alongside US, while the EASL–EORTC guidelines express concerns about cost-effectiveness and suggest relying on US alone for routine surveillance. Despite these differences, all three guidelines agree that US has limitations, particularly in obese patients or those with advanced cirrhosis where additional imaging modalities such as CEUS, CT, or MRI may be required for a more accurate assessment when the initial findings are inconclusive.

The inclusion of biomarkers such as AFP, DCP, and other markers could further enhance diagnostic accuracy, especially in high-risk patients or those with inconclusive imaging. The APASL guidelines’ approach of adjusting the AFP cut-off values based on population-specific factors offers a potential model for future guidelines. Furthermore, the growing use of liquid biopsy and the integration of AI into clinical practice hold promise for refining risk stratification and personalizing surveillance strategies.

An emerging concern in HCC surveillance is the rising incidence of hepatocellular carcinoma in patients with non-cirrhotic NAFLD, a group that is often excluded from the current protocols. Growing evidence indicates that non-cirrhotic NAFLD can lead to HCC, highlighting the need to broaden the surveillance criteria to include these patients as their risk may be substantial if left unmonitored. Similarly, those with baseline non-cirrhotic severity or post-viral control, such as those achieving viral suppression in hepatitis B or C, continue to carry a non-negligible risk of HCC. This population warrants a tailored approach to avoid high rates of missed diagnoses and ensure effective early detection.

For these emerging and lower-risk populations, a risk-adjusted strategy that leverages specific clinical, genetic, and molecular factors can provide a clearer and more targeted framework, helping to prevent unnecessary interventions and optimize resource allocation. For instance, setting distinct surveillance thresholds based on risk factors that are unique to non-cirrhotic NAFLD and post-viral control may enable more precise risk stratification. Additionally, further research into the mechanistic pathways and progression of HCC within these groups is essential to refine surveillance protocols. These recommendations aim to support the development of evidence-based criteria that align HCC surveillance with the principles of precision medicine and address the evolving needs of diverse patient populations.

## Figures and Tables

**Table 1:** High-risk groups for which HCC surveillance is recommended as per APASL guidelines. Reproduced with permission from [14], Hepatology International; published by Springer, 2017. This work is distributed under the terms of the Creative Commons Attribution 4.0 International License (http://creativecommons.org/licenses/by/4.0/ (accessed on 6 September 2024)). Only minor formatting adjustments were made.

High-Risk Group	Hepatocellular Carcinoma (HCC) Risk (per Year)
Cirrhotic hepatitis patients	
Hepatitis B Virus (HBV)	3–5%
Hepatitis C Virus (HCV)	2–7%
Non-alcoholic steatohepatitis (NASH)	2–4%
Autoimmune disorders	
Primary biliary cholangitis (PBC)	2–3%
Autoimmune hepatitis	Unknown
Genetic conditions	
Genetic hemochromatosis	Unknown, but probably > 1.5%
Alpha-1 antitrypsin (A1AT) deficiency	Unknown, but probably > 1.5%
Other etiologies	
Chronic HBV carriers Non-cirrhotic (HBsAg positive)	
Asian females > 50 years	0.3–0.6%
Asian males > 40 years	0.4–0.6%
Africans aged > 20 years	Not available
History of HCC in the family	Not available

**Table 2:** Comparison of HCC surveillance recommendations across the APASL, AASLD, and EASL guidelines.

Key Guideline Aspects	Asian Pacific Association for the Study of the Liver (APASL) [14]	American Association for the Study of Liver Diseases (AASLD) [15]	European Association for the Study of the Liver and European Organization for Research and Treatment of Cancer (EASL–EORTC) [13]
Surveillance protocol	Biannual surveillance at six-month intervals recommended, using a combination of abdominal ultrasound (US) and serum alpha-fetoprotein (AFP).	Semi-annual surveillance at six-month intervals recommended, using a combination of abdominal US and serum AFP. Outreach interventions are also advised to enhance both provider and patient adherence to regular screening.	Regular surveillance every six months recommended, using abdominal US.
Patient selection criteria	Cirrhotic patients, those with associated genetic conditions (hemochromatosis or A1AT deficiency), autoimmune liver disorders (PBC or autoimmune hepatitis), and NASH.	Cirrhotic patients (Child-Pugh A and B) and Child-Pugh C patients awaiting liver transplant.	Cirrhotic patients (Child-Pugh A and B) and Child-Pugh C patients awaiting liver transplant. Surveillance for non-cirrhotic patients (chronic HBV classified as intermediate/high risk for HCC or advanced fibrosis) is also recommended based on individual risk assessment.
Preferred screening tools	Abdominal US and serum AFP.	Abdominal US and serum AFP.	Abdominal US.
Follow-up criteria and referral	Follow-up and referral based on imaging characteristics found during screening (iso- or hypovascularity in the arterial phase or hypervascularity without portal venous washout). Further evaluation includes additional imaging with computed tomography (CT), magnetic resonance imaging (MRI), or contrast-enhanced US (CEUS).	Follow-up and referral procedures based on US visualization, presence of liver lesions, and AFP levels. For subcentimeter lesions, repeat US and AFP in 3–6 months is recommended. For lesions ≥ 1 cm or those with elevated AFP levels, additional imaging with CT or MRI recommended. For patients with AFP levels ≥ 200 ng/mL but no detectable mass, positron emission tomography-CT (PET-CT) is recommended.	For nodules < 1 cm, continued monitoring at intervals of ≤4 months during the first year should be conducted. For lesions ≥ 1 cm, additional imaging with CT or MRI is recommended. Liver biopsy is recommended if imaging modalities are inconclusive. Referral to specialized centers for suspicious nodules is emphasized.

**Table 3:** Summary of imaging modalities in HCC surveillance.

Key Aspects	US	CEUS	CT	MRI
Primary use	Screening in at-risk populations [3,14].	Characterization of US-detected liver nodules [3,14].	Diagnostic tool when US findings are suspicious or inconclusive [3,21].	Diagnostic tool when US findings are suspicious or inconclusive [3,21].
AASLD recommendations	First-line screening tool, recommended every 6 months in high-risk populations [21].	Not recommended as a diagnostic tool due to concerns over false positives [21].	Reserved for cases with inadequate US or suspicious lesions, not recommended for routine screening [21].	Reserved for cases with inadequate US or suspicious lesions, not recommended for routine screening [21].
EASL–EORTC recommendations	First-line screening tool, recommended every 6 months in high-risk populations [13].	Not routinely recommended, considered for further characterization in specific cases [13].	Used for further evaluation of suspicious US findings, not routinely recommended for screening [13].	Used for further evaluation of suspicious US findings, preferred over CT in some cases due to higher sensitivity [13].
APASL recommendations	First-line screening tool, recommended every 6 months in high-risk populations [14].	Supported for characterizing indeterminate US findings, considered cost-effective [14].	Used for further evaluation in high-risk patients, recommended when US findings are indeterminate [14].	Used for further evaluation in high-risk patients, recommended when US findings are indeterminate [14].
Sensitivity	40–81%, varies by operator skill and patient factors [3].	Comparable to CT/MRI, pooled sensitivity: 73.6–84.4% [3,14].	68–91%, depending on lesion size and contrast enhancement [3].	77–100%, Gd-EOB-DTPA-enhanced MRI is particularly sensitive for small tumors [3].
Specificity	80–100% [3].	Comparable to CT/MRI, pooled specificity: 83.6–89.3% [3,14].	>95%, especially when identifying hallmark features like arterial enhancement [14].	>95%, particularly effective with contrast-enhanced imaging [3].
Cost-effectiveness	High, low cost, widely available [14].	High, lower cost than CT/MRI with no ionizing radiation [14].	Moderate, higher cost, and involves ionizing radiation [3].	Lower than US, but high diagnostic yield and cost-effective for detailed imaging [14].
Radiation exposure	None [3].	None [3].	Yes, involves ionizing radiation which is a concern for long-term surveillance [3].	None [3].
Contrast agent considerations	Not applicable.	Uses microbubble contrast agents, non-nephrotoxic, no ionizing radiation [14].	Uses iodinated contrast agents., risk for patients with renal impairment [3].	Uses gadolinium-based contrast agents, risk in patients with severe renal impairment [3].

**Table 4:** Summary of current and emerging biomarkers in HCC surveillance.

Biomarker	Description	Use in HCC Screening	Sensitivity/Specificity	Included in Guidelines
AFP	A glycoprotein produced by the fetal liver, elevated levels (>200 ng/mL) may indicate HCC [27].	Most commonly used biomarker, used as an adjunct to imaging to identify patients needing further evaluation [27].	Sensitivity: 39–64%, specificity: 76–97% [27].	AASLD, EASL, APASL [13,14,15].
AFP-L3	A glycoform of AFP that is more specific for HCC. Elevated levels are associated with a higher risk of HCC [14].	More specific than total AFP, useful in differentiating HCC from benign liver conditions in patients with elevated total AFP levels [14].	Sensitivity: 49–62%, specificity: 90% [14].	APASL [14].
DCP	Also known as PIVKA-II, an abnormal form of prothrombin. Elevated in HCC patients [23].	Useful in detecting HCC in patients with normal AFP levels, often used in conjunction with other markers in Asia [23].	Sensitivity: 34–40%, specificity: 81–98% [23].	APASL [14].
OPN	A glycoprotein involved in bone metabolism, elevated in several cancers, including HCC [28].	Potentially useful in distinguishing between HCC and benign liver diseases, could be used alongside other markers like AFP [28].	Sensitivity: 49%, specificity: 72% [28].	Not specified.
GPC3	A heparan sulfate proteoglycan overexpressed in HCC patients [29].	Found in serum and tissue samples, high specificity for HCC, often used in conjunction with immunohistochemistry for HCC diagnosis [29].	Sensitivity: 55%, specificity: >95% [29].	Not specified.
GP73	A membrane protein expressed in hepatocytes, levels are elevated in HCC patients [30].	Considered more sensitive than AFP in some studies, potentially useful for early detection of HCC, still under investigation and not yet widely adopted in clinical practice [30].	Sensitivity: 62–79%, specificity: 62–88% [30].	Not specified.

## Data Availability

No new data were created or analyzed in this study. Data sharing is not applicable to this article.

## Supplementary Materials

The following supporting information can be downloaded at: https://www.mdpi.com/article/10.3390/cancers16233933/s1, Table S1. Quality Assessment of Reviewed Studies. Figure S1. PRISMA Chart.
