# Supplementary material for: Hepatocellular Carcinoma Surveillance Strategies: Major Guidelines and Screening Advances
Source: Cancers (Basel). 2024 Nov 24;16(23):3933. doi: 10.3390/cancers16233933 (PMC11640160; doi:10.3390/cancers16233933)
Supplement: Supplementary file 1 [file cancers-16-03933-s001.zip › cancers-3279362-supplementary.pdf]

cancers

MDPI

Supplementary Materials

Literature Search Strategy
A systematic literature search was conducted using electronic databases, including PubMed, Embase, and Google Scholar, covering studies published from January 2008 to April 2024. The search included the following keywords and phrases: "hepatocellular carcinoma," "HCC surveillance," "biomarkers," "ultrasound," "APASL," "AASLD," "EASL," and "HCC early detection." Reference lists of relevant articles were also manually reviewed to identify additional studies.

Inclusion Criteria: Peer-reviewed studies published in English that discuss HCC surveillance guidelines, imaging modalities, biomarkers, and advancements in screening techniques were included. Studies addressing high-risk populations, surveillance strategies, or the limitations and effectiveness of various imaging and biomarker methodologies were prioritized.

Exclusion Criteria: Non-English publications, case reports, opinion pieces, and studies with low-quality assessments were excluded.

PRISMA Flow Diagram (Supplementary Figure S1)
The PRISMA flow diagram (**Figure S1**) summarizes and provides a visual representation the study selection process of studies included in this review. Out of the initial records identified through database and manual searches, duplicates were removed, and titles/abstracts were screened. Studies that did not meet the eligibility criteria based on relevance, quality, or language were excluded. Full-text articles were further assessed, with final inclusion resulting in 50 studies for qualitative synthesis.

Quality Assessment of Reviewed Studies (Supplementary Table S1)
A quality assessment was performed on each study using criteria from the Newcastle-Ottawa Scale for cohort studies and other appropriate scales for systematic reviews. The reviewed studies are listed in **Table S1**, which includes the study citation, design, quality score, and key findings.

Table S1. Quality Assessment of Reviewed Studies

Study Citation	Study Design	Quality Score	Key Findings
Wen, N. et al., 2022	Review	High (8/9)	Comparison of global HCC management guidelines; noted gaps in non-cirrhotic NAFLD surveillance.
Sung, H. et al., 2021	Cohort Study	Moderate (7/9)	GLOBOCAN statistics on HCC incidence and mortality; noted increase in HCC cases associated with NAFLD.
Asafo-Agyei, K.O. & Samant, H., 2024	Review	High (8/9)	Overview of HCC risk factors and global disease burden; helpful context for guideline differences.

Bajestani, N. et al., 2024	Cohort Study	Moderate (6/9)	Assessed efficacy and safety of combined locoregional therapy and immunotherapy for HCC.
Ito, K. et al., 2020	Systematic Review	High (9/9)	Compared HCC treatment algorithms across major guidelines; insights into surveillance tool variations.
Bray, F. et al., 2024	Cohort Study	High (8/9)	Updated GLOBOCAN data; highlighted emerging trends in global HCC incidence and survival outcomes.
Makary, M.S. et al., 2021	Case Series	Moderate (7/9)	Evaluated outcomes of locoregional therapies for HCC; provided insights into treatment safety.
Frenette, C.T. et al., 2019	Practical Guideline	High (8/9)	Practical guidance on HCC screening in high-risk populations; proposed cost-effective approaches.
Jipa, A.M. & Makary, M.S., 2024	Review	High (8/9)	Examined recent advancements in locoregional therapies for hepatobiliary tumors, including HCC.
Huang, D.Q. et al., 2023	Narrative Review	High (8/9)	Discussed barriers to HCC surveillance and effects of shifting disease etiology.
Makary, M.S. et al., 2020	Review	High (8/9)	Analysis of locoregional therapy approaches; proposed updates to HCC management strategies.
Qurashi, M. & Sharma, R., 2024	Review	Moderate (7/9)	Challenges and solutions in HCC surveillance in the UK; recommended tailored approaches.
Galle, P.R. et al., 2018	Clinical Practice Guidelines	High (9/9)	EASL guidelines for HCC management; focused on biomarker use and imaging in high-risk populations.
Omata, M. et al., 2017	Clinical Practice Guidelines	High (9/9)	APASL guidelines; addressed surveillance tailored for Asian populations with HCC risk.
Singal, A.G. et al., 2023	Practice Guidance	High (9/9)	AASLD guidance on HCC prevention, diagnosis, and surveillance; emphasized biomarker usage.

Cho, Y. et al., 2023	Review	High (8/9)	Comparison of Asian HCC guidelines; discussed surveillance strategy variations across regions.
Foerster, F. & Galle, P.R., 2019	Review	Moderate (7/9)	Compared international HCC guidelines, emphasizing management and surveillance differences.
Criss, C. et al., 2023	Diagnostic Imaging Review	High (8/9)	Evaluated imaging modalities in HCC diagnostics; focused on state-of-the-art imaging techniques.
Pascual, S. et al., 2019	Systematic Review	Moderate (7/9)	Systematic review of HCC surveillance modalities; assessed diagnostic accuracy and cost-effectiveness.
Kudo, M., 2021	Review	Moderate (6/9)	Proposed systemic therapy-first approach for certain HCC patients, with potential for conversion to curative treatment.
Marrero, J.A. et al., 2018	Practice Guidance	High (9/9)	AASLD guidelines on HCC management; covered diagnostic criteria, staging, and imaging approaches.
Debes, J. et al., 2021	Review	High (8/9)	Overview of serum biomarkers in HCC; discussed their role in early detection and prognosis.
Lok, A.S. et al., 2010	Cohort Study	Moderate (7/9)	Assessed effectiveness of AFP and DCP in early HCC detection among cirrhotic patients.
Kudo, M. et al., 2014	Practice Guidelines	High (9/9)	Japanese guidelines on HCC management; included specific biomarker and imaging recommendations.
Zhao, S. et al., 2020	Meta-analysis	High (8/9)	Diagnostic value of combining biomarkers for HCC detection; demonstrated improved sensitivity.
Khan, I.M. et al., 2021	Cohort Study	Moderate (6/9)	Evaluated a novel biomarker panel for early HCC detection in cirrhotic patients.
Hanif, H. et al., 2022	Review	Moderate (7/9)	Reviewed applications and limitations of AFP for HCC diagnosis; emphasized

			need for alternative biomarkers.
Yan, Z. et al., 2023	Review	Moderate (7/9)	Discussed osteopontin as a potential biomarker in HCC diagnosis and treatment strategies.
Guo, M. et al., 2020	Review	High (8/9)	Overview of glypican-3 in HCC diagnostics; discussed potential for targeted therapies.
Wang, Y. & Wan, Y.-J.Y., 2020	Review	High (8/9)	Discussed roles of Golgi Protein 73 and glypican-3 in HCC and other cancers.
Pouwels, S. et al., 2022	Review	Moderate (6/9)	Reviewed pathophysiology and clinical management of NAFLD and its link to HCC.
Huang, D.Q. et al., 2021	Epidemiological Review	High (8/9)	NAFLD-related HCC epidemiology; discussed risk factors and trends.
Forner, A. et al., 2018	Review	High (8/9)	Comprehensive review of HCC; highlighted biomarkers and imaging for surveillance.
Patil, M. et al., 2013	Case Report	Low (5/9)	Report on elevated AFP levels without HCC; discussed challenges in AFP interpretation.
Bai, D.-S. et al., 2017	Cohort Study	Moderate (7/9)	Correlation between AFP level and HCC prognosis; implications for treatment decisions.
Giannini, E.G. et al., 2012	Cohort Study	Moderate (7/9)	Found AFP to have limited prognostic value in small HCC identified during surveillance.
Pan, Y. et al., 2020	Review	Moderate (7/9)	Discussed current status and future potential of various HCC biomarkers.
Morishita, A. et al., 2021	Review	High (8/9)	Reviewed microRNAs in HCC pathogenesis; potential as diagnostic and prognostic biomarkers.
Kopystecka, A. et al., 2023	Review	Moderate (6/9)	Discussed the use of ctDNA in HCC diagnosis and monitoring; emerging tool for liquid biopsy.
Ginghina, O. et al., 2022	Review	Moderate (6/9)	Explored AI applications in liquid biopsy for cancer diagnosis; included potential for HCC.

Manea, I. et al., 2023	Review	Moderate (6/9)	Overview of liquid biopsy in early HCC detection; focused on advantages and limitations.
Jiménez Pérez, M. & Grande, R.G., 2020	Review	Moderate (7/9)	Application of AI in HCC diagnosis and treatment; highlighted benefits and challenges of AI integration.
Cholankeril G, El-Serag HB. Semin Liver Dis. 2023;43(1):89–99.	Review	High (8/9)	Addressed current challenges in HCC surveillance for NAFLD, emphasizing need for tailored approaches.
Plaz Torres MC, et al. Cancers (Basel). 2020;12(6):1422.	Review	Moderate (7/9)	Discussed selective vs. universal HCC surveillance for NAFLD patients, highlighting pros and cons.
Innes H, Nahon P. J Hepatol. 2023;79(5):1332–1337.	Statistical Analysis	High (9/9)	Provided statistical insights on using risk models for surveillance decision-making.
Rich NE, et al. Semin Liver Dis. 2017;37(4):296–304.	Review	Moderate (7/9)	Examined the issue of overdiagnosis in HCC surveillance, particularly in low-risk populations.
Demirtas CO, Brunetto MR. World J Gastroenterol. 2021;27(33):5536–5554.	Review	High (8/9)	Discussed personalized surveillance for HCC in chronic viral hepatitis patients.
Lee YT, et al. Hepatology. 2023;78(1):319–362.	Review	High (8/9)	Proposed early detection biomarkers for precision HCC screening.
Singal AG, et al. J Hepatol. 2023;79(1):226–239.	White Paper	High (9/9)	ILCA white paper on HCC risk stratification, emphasizing need for personalized surveillance.
Fujiwara N, et al. J Hepatol. 2018;68(3):526–549.	Review	High (8/9)	Reviewed risk factors and prevention methods for HCC within the context of precision medicine.

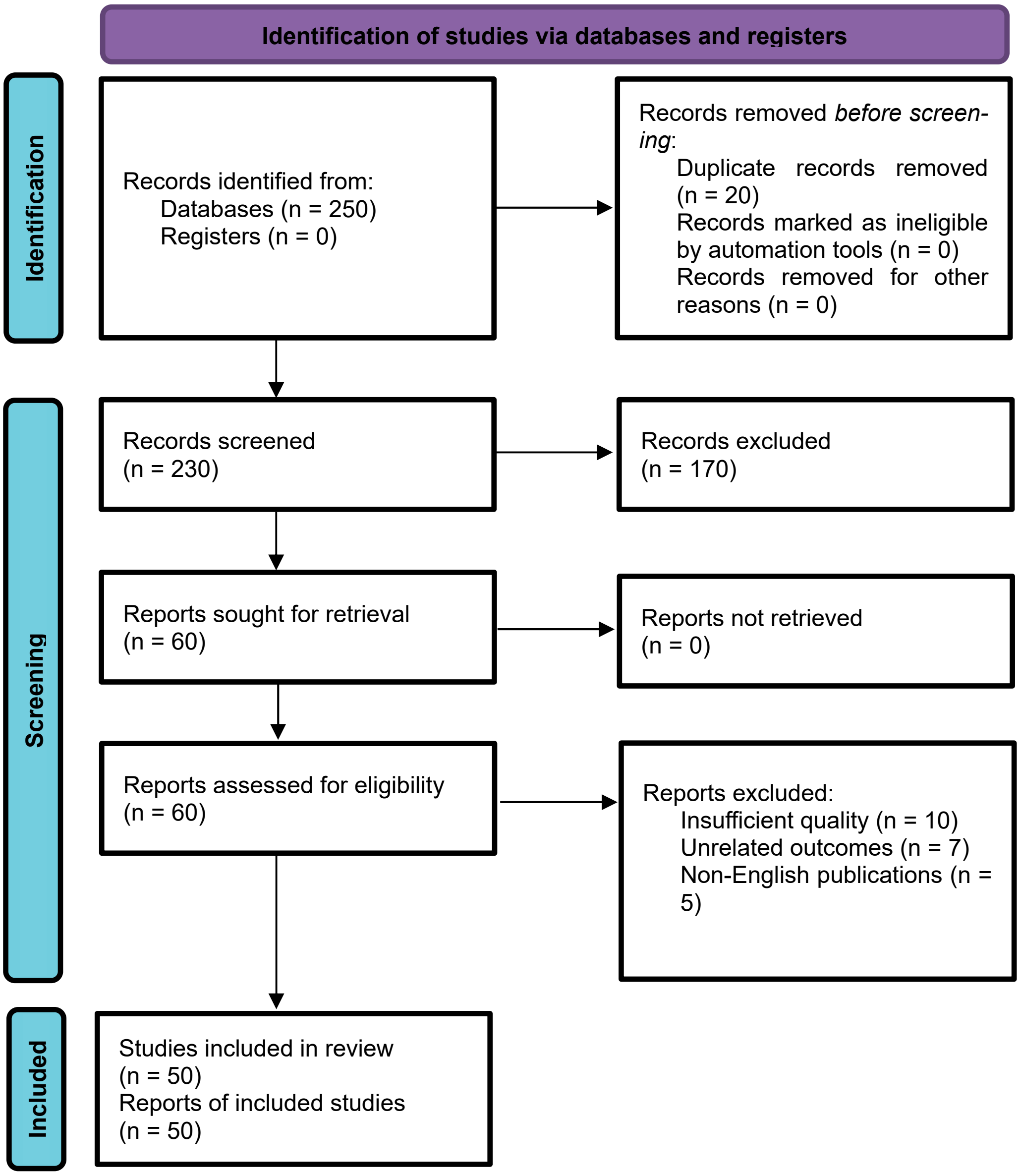

Figure S1. PRISMA Chart

Disclaimer/Publisher's Note: The statements, opinions and data contained in all publications are solely those of the individual author(s) and contributor(s) and not of MDPI and/or the editor(s). MDPI and/or the editor(s) disclaim responsibility for any injury to people or property resulting from any ideas, methods, instructions or products referred to in the content.
